# Persistence of the Probiotic *Lacticaseibacillus rhamnosus* Strain GG (LGG) in an In Vitro Model of the Gut Microbiome

**DOI:** 10.3390/ijms232112973

**Published:** 2022-10-26

**Authors:** Karley K. Mahalak, Jenni Firrman, Jamshed Bobokalonov, Adrienne B. Narrowe, Kyle Bittinger, Scott Daniel, Ceylan Tanes, Lisa M. Mattei, Wei-Bin Zeng, Jason W. Soares, Masuko Kobori, Johanna M. S. Lemons, Peggy M. Tomasula, LinShu Liu

**Affiliations:** 1 Dairy and Functional Foods Research Unit, Eastern Regional Research Center, Agricultural Research Service, United States Department of Agriculture, 600 E Mermaid Lane, Wyndmoor, PA 19038, USA; 2 Division of Gastroenterology, Hepatology, and Nutrition, The Children’s Hospital of Philadelphia, Philadelphia, PA 19104, USA; 3 Department of Mathematics, University of Louisville, Louisville, KY 40292, USA; 4 Bioprocessing and Bioengineering Group, US Army Combat Capabilities Development Command Soldier Center (CCDC-SC), Natick, MA 01760, USA; 5 Institute of Food Research, National Agriculture and Food Research Organization, Tsukuba 305-8642, Ibaraki, Japan

**Keywords:** probiotics, LGG, gut microbiome, 16S rRNA sequencing, short-chain fatty acids

## Abstract

The consumption of probiotics is widely encouraged due to reports of their positive effects on human health. In particular, *Lacticaseibacillus rhamnosus* strain GG (LGG) is an approved probiotic that has been reported to improve health outcomes, especially for gastrointestinal disorders. However, how LGG cooperates with the gut microbiome has not been fully explored. To understand the interaction between LGG and its ability to survive and grow within the gut microbiome, this study introduced LGG into established microbial communities using an in vitro model of the colon. LGG was inoculated into the simulated ascending colon and its persistence in, and transit through the subsequent transverse and descending colon regions was monitored over two weeks. The impact of LGG on the existing bacterial communities was investigated using 16S rRNA sequencing and short-chain fatty acid analysis. LGG was able to engraft and proliferate in the ascending region for at least 10 days but was diminished in the transverse and descending colon regions with little effect on short-chain fatty acid abundance. These data suggest that the health benefits of the probiotic LGG rely on its ability to transiently engraft and modulate the host microbial community.

## 1. Introduction

The probiotic *Lacticaseibacillus* (formerly *Lactobacillus*) *rhamnosus* strain GG (LGG) has been approved by the FDA for use as a dietary supplement in the United States and is also used for therapeutic purposes in many other countries throughout Asia and Europe [1,2]. Indeed, it has been reported that LGG is able to prevent, treat, or alleviate some symptoms associated with obesity, depression, cardiovascular diseases, and gastrointestinal disorders [1,3,4]. A commonly occurring claim is that LGG and other probiotics can restore a dysbiotic microbiome by recovering the diversity and richness typical of healthy gut microbiota [5]. Dysbiosis is associated with many diseases, including chronic autoimmune inflammatory diseases [6,7], Crohn’s disease, chronic diarrhea of the lower bowel [8,9,10,11,12,13,14], cardiovascular diseases [15,16], diabetes, and obesity [17,18,19], as well as stress and anxiety [20,21,22,23,24]. In contrast, some clinical trials and in vivo experiments on animal models have reported probiotics to be ineffective or to even have contradictory outcomes and ascribing side effects with LGG use, such as brain fogginess [25], inefficiency as treatment of acute gastroenteritis [26], ileal pouch inflammation [27], and increased incidence of bacteremia, endocarditis, and sepsis-induced dysbiosis [3,28].

Modern advances in metagenomics and chemical instrumental analysis have recently made it possible to interrogate the effect of probiotics on the gut microbiota more closely [29,30]. As research capabilities evolve, it is important to identify the extent of the impact of probiotics, and especially of LGG. It is essential to have a large volume of well-designed, and well-conducted trials that include but are not limited to knowing the case-specified dosage, the residence time of probiotics in the gastrointestinal tract (GIT), and the interactions between the probiotics with the microbe of the host GIT.

In the present research, the survival and growth of LGG in mature gut microbial communities were investigated. The in vitro gut microbial communities were established within a simulator of the human intestinal microbial ecosystem (SHIME^®^) [31,32,33], using human fecal samples from three healthy donors. The experiments were performed under conditions mimicking the GIT’s physiological kinetics, without interferences from the mammalian milieu, thus ensuring that the interaction between the exogenous LGG and the host bacteria was the sole variable. The changes to the community composition longitudinally within the colon in response to LGG were determined using 16S rRNA marker gene sequencing. The short-chain fatty acids produced by the microbial community were examined using gas chromatography-mass spectrometry (GC-MS). Together, these results demonstrated the persistence of the probiotic LGG within the gut microbiota as well as its effect on the endogenous gut microbial community in terms of structure and function.

## 2. Results and Discussion

### 2.1. Survival and Growth of LGG in a Multi-Compartment In Vitro Model of the Colon

In the present study, the survival/growth profile of LGG was examined in vitro. This was done using the SHIME^®^ platform, which is a multi-compartment, in vitro apparatus that is designed to mimic the ascending (AC), transverse (TC), and descending (DC) colon regions of the GIT [32,34]. This experiment was performed in triplicate, using fecal inoculum from 3 separate individuals, indicated as biological replicates 1–3 (BR1, BR2, and BR3).

**Figure 1:**
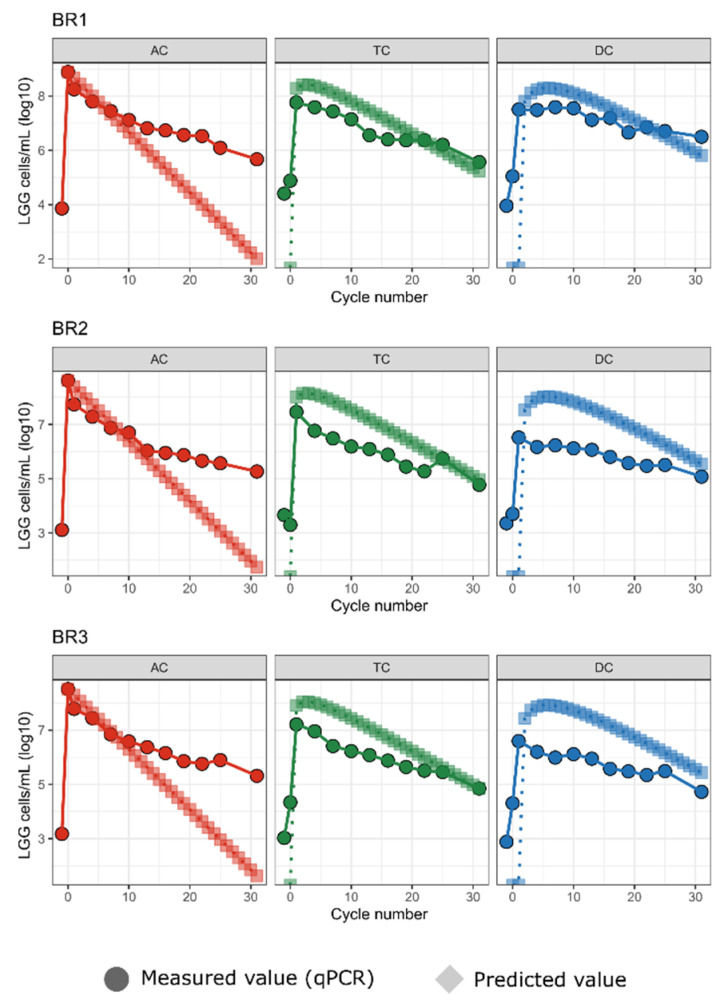
LGG in bioreactors persists and replicates in ascending colon reactors. For each of three bioreplicates, predicted LGG cell counts under a no-growth model vs. measured (qPCR) LGG cell counts are shown for each bioreactor (AC, TC, DC) over 31 transit cycles. Predicted counts are shown as circles.

**Figure 2:**
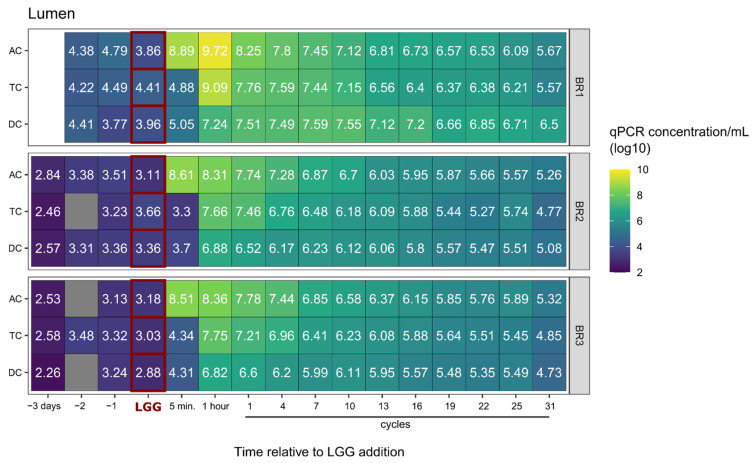
LGG cell counts in lumenal samples. Heatmap showing qPCR LGG cell counts in lumenal samples for each of the three bioreplicates (BR1, BR2, BR3). Prior to the LGG addition to the system, there was negligible LGG presence, but LGG persists in the reactors over the course of 31 cycles. Cell counts are displayed in log scale.

**Figure 3:**
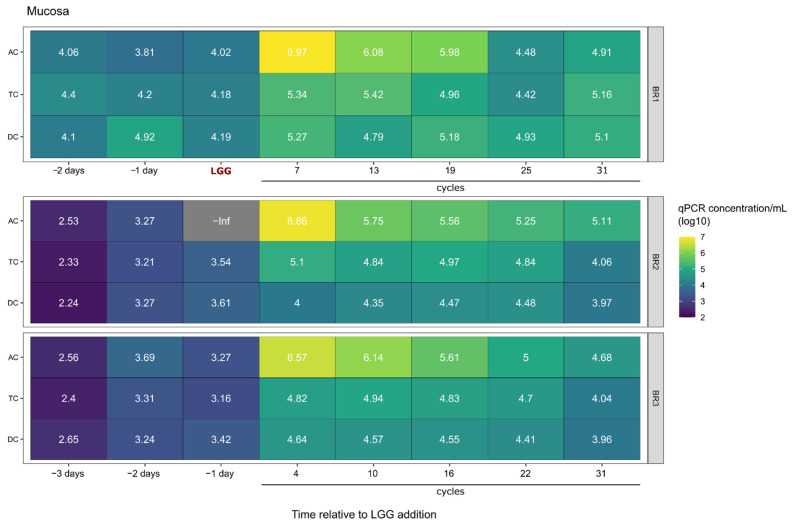
LGG Cell counts in mucosal samples. Heatmap showing qPCR LGG cell counts in mucosal samples for each of three bioreplicates (BR1, BR2, BR3). Prior to LGG addition to the system, there was negligible LGG presence, but LGG persisted in the reactors over the course of 31 cycles.

The endogenous concentration of LGG in the 3 simulated colons ranged from 1.3 × 10^3^ to ~8 × 10^4^ cells/mL. BR1 consistently exhibited a 10-fold higher concentration than either BR2 or BR3. Upon the addition of the exogenous LGG to the SHIME, the LGG percentage in the communities within the colon regions was measured. Immediately after the increase in LGG following its addition to the AC, all three simulated colons exhibited an initial sharp decrease in LGG population in the luminal phase of all regions (AC, TC, DC) during the first 7–10 feeding cycles, followed by a slower yet continuous reduction through the conclusion of the experiment (Figure 1). At the end of the experiment, the measured LGG contents in the AC, TC, and DC regions of each community were similar, being 7-fold to 540-fold (1–2 log) higher than that in the original community (Figure 2). In this study, the LGG’s survival/growth profiles were also donor-dependent. For BR2 and BR3, the measured LGG content was reduced in the sequence of AC > TC > DC over the experimental period. For BR1, the LGG content in the AC region was higher than TC and DC before feeding cycle 7, but after that LGG content in DC was higher than in AC and TC (Figure 1, Figure 2 and Figure 3).

Next, we measured the ability of LGG to colonize the mucin carriers added to each simulated colon region to represent the mucosal surface within the GIT. As shown in Figure 3, the LGG content in the mucin carriers was low, 1–2 log less than that measured in the luminal phase (Figure 2 and Figure 3). Displaying a similar trend to that seen in the luminal phase, the LGG content in mucin carriers in all colonic regions decreased as the fermentation time increased; however, the reduction rate was milder compared to the luminal phase, with only a 1 log reduction over the entire experimental period. At the end of the experiment, the LGG content in mucin carriers remained higher than that measured in the original communities, increasing from 1.5- to 27-fold from pre- to post-experimental time points with the exception of the AC region of BR1 which increased from zero to 1.28 × 10^5^ cells/mL.

Since the endogenous LGG concentrations of the 3 bioreplicates (BR1, BR2, BR3) were constant and considered as the baseline, this amount was subtracted from the measured amount to provide the amount of LGG that survived or proliferated following probiotic treatment. To confirm it, we compared the measured number with theoretically calculated LGG content based on an equation calculating the decrease in administrated LGG over time due to dilution from each feeding cycle (Figure 1).

### 2.2. LGG Persistence and Growth Versus Transient Presence over Time

Following a single addition of LGG to the system, unless the cells were able to multiply, it was assumed that the cell count would decrease via dilution and transit with the subsequent cycles of defined medium addition and movement of mass through and out of the SHIME^®^ system. This change was mathematically calculated using a mechanistic model as described below. This model assumed that there was no growth and loss of the exogenous LGG occurred after the cells were added to the SHIME^®^, cells bound to the mucin carriers could be ignored (the total mucin gel and the LGG content per mL mucin are low), and that the change in bacterial number at each time point in each bioreactor co-occurred with feedings and transit one region to the next three times a day. The change in LGG content was calculated using a mechanistic model as follows:

Let r(t) be the content percentage of the incoming transfer at time *t*. Over the time interval Δt, the amount of content transferred would be approximately mr(t)Δt, and it follows that the total amount of content transferred would be:

(1) $$\int_{0}^{t_{0}}mr\left(t\right)dt$$

By the mean-value theorem [35], we have:

(2) $$r_{0}\left(t_{0}-0\right)=\int_{0}^{t_{0}}r\left(t\right)dt$$

where $r\left(0\right)\;\leq r_{0}\leq \;r\left(t_{0}\right)$ for some $r_{0}$, and the constant *m* is cancelled. The equation for the content in a bioreactor is:

(3) $$M_{1}\int_{0}^{t_{0}}mr_{1}\left(t\right)dt+M_{2}\int_{0}^{t_{0}}mr_{2}\left(t\right)dt=\left(M_{1}+M_{2}\right)\int_{0}^{t_{0}}mr\left(t\right)dt$$

It can be reduced to:

(4) $$M_{1}r_{1}+M_{2}r_{2}=\left(M_{1}+M_{2}\right)r$$

where $r_{1}$ is the incoming content percentage in the amount $M_{1}$, and $r_{2}$ is the content percentage in the amount $M_{2}$ already in the bioreactor, and the basic formula is then:

$$r=\frac{M_{1}r_{1}+M_{2}r_{2}}{M_{1}+M_{2}}$$

Using the above equation, we calculated the expected number of cells in this no-growth, washout condition based on the starting LGG qPCR values at the time of LGG addition to the system for each experiment. Predicted cell counts were initially highest in the AC, followed by a peak in the TC as the AC was diluted with the thrice-daily addition of LGG-free feed and cells moved to the TC, followed by a similar peak in the DC, with all three reactors ultimately approaching zero at 30 cycles (Figure 1). We compared these expected cell counts with the actual cell counts as measured by qPCR.

In all three experiments, the measured LGG cell counts (Figure 1, circles) deviated from the theoretical no-growth concentrations (Figure 1, diamonds). In the AC, the measured counts were level with and eventually exceeded the expected counts. In contrast, in the TC and the DC, the curves of the measured LGG tracked the shape of the theoretical curves but were lower in magnitude, with the measured LGG beginning to reach the theoretical values only beginning at cycle 25. The higher-than-expected concentration in the AC and lower-than-expected concentration in the TC and DC suggest that the exogenous LGG was persisting and possibly growing in the AC, and that these cells were not moving to the TC and DC at the expected rates. Alternatively, the LGG was persisting and growing in the AC and was also moving to the TC and DC at the expected rates, but was experiencing higher mortality in those regions, resulting in lower-than-expected counts.

There are three possible endpoints for exogenous LGG cells after entering a mature gut microbial community in the SHIME^®^: remain viable but not proliferate, remain viable and proliferate, or die. Only when the probiotic grows faster than the sum of the LGG that is dead and transported out can the LGG in the system persist in the model gut system. It is possible that after the LGG acclimates to the environmental fluctuations caused by the three times per day feeding regimen, it will start growing. Indeed, the LGG in the AC region appears to have benefitted from the rich nutrients present there since the measured amount was higher than what was predicted if they had just washed out. As LGG was transported to the TC and DC regions, where comparatively fewer nutrients were available and competition with other bacteria was higher, survival was further challenged. Although at the end of the experiment, the LGG concentration in the three regions of the three communities was 1–2 log higher than that measured before the exogenous LGG was added, the differences between the measured and predicted concentrations declined in the TC and DC regions. In our hands, the content of the LGG associated with the mucin carriers was found to be 1–2 log lower than the content in the luminal phase and thus it is hard to say if it plays any role in prolonging the residence in the GIT [36,37]. More research on optimizing the in vitro mucin model is still needed. It is thought to be extremely difficult for foreign microbes, such as exogenously added probiotics, to engraft in an established symbiotic ecosystem because of vigorous competition for niche and nutrients, especially against the endogenous LGG. It is expected that eventually most exogenously added microbes will be removed by the mass flow from the daily feeding cycles [37,38,39].

### 2.3. Impact on the Host Microbial Community

**Figure 4:**
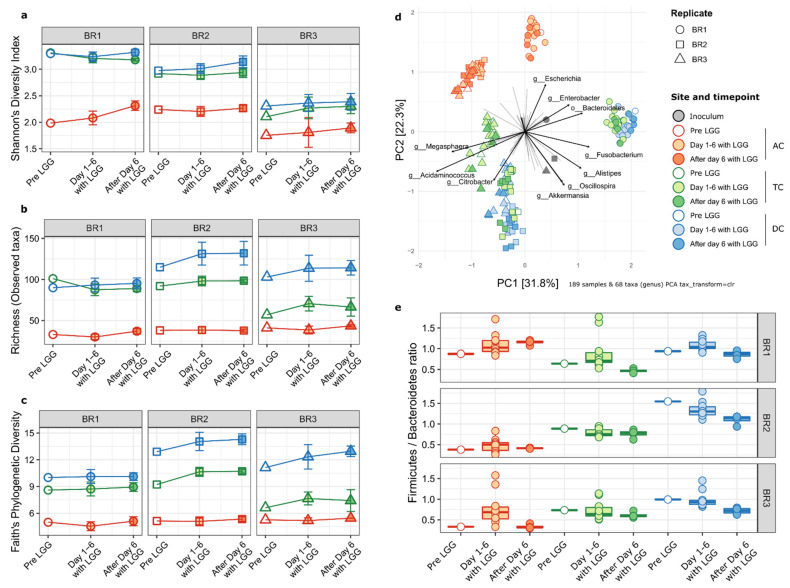
Community diversity and F/B ratio. Alpha-diversity metrics display consistent patterns regardless of LGG addition. For all three bioreplicates, the TC and DC are significantly higher in all sites while within each site, diversity does not change over time relative to LGG addition. (**a**) Shannon’s diversity index, (**b**) richness (observed ASV counts) (**c**) Faith’s phylogenetic distance. Colon regions (reactors) are shown by colors. Red, AC; Green; TC; Blue, DC. (**d**) PCA biplot of total ASV abundances showing samples (points) and taxa (arrows). Points are colored by site and time relative to LGG addition, with shapes representing the bioreplicates. The top ten taxa driving the distribution of samples are shown as arrows with the length of arrows representing the PCA loadings (strength of association). TC and DC samples are more similar to each other than to AC samples. BR2 and BR3 samples are more similar to each other than to BR1 samples. (**e**) Boxplots showing Firmicutes to Bacteroidetes ratio for samples. Boxes show first to third quartiles and median. Individual sample ratios are shown as points.

Using 16S rRNA marker gene sequencing, we characterized the microbial community structure and composition for each colon region of the bioreplicates prior to and following LGG addition. First, we measured the impact of LGG on alpha-diversity of the established gut microbial community, in terms of Shannon’s diversity index, species richness (observed ASVs), and Faith’s phylogenetic diversity (Faith’s P.D.) index. Since the experimental addition of LGG to the community would be expected to increase these values, LGG ASVs were removed from the feature table before calculating alpha diversity metrics. The Shannon index reflects both the abundance and presence of the species in each sample, with a higher number indicating a higher diversity. With the exception of the AC region samples from BR1, there were no significant differences in the Shannon index between time points of any colonic regions of the three bioreplicates (Figure 4a). However, for all three bioreplicates, both the TC and DC were significantly more diverse than the AC at all time points. This was similar to previous reports that found the AC community to have lower diversity in the SHIME^®^ system [32,34]. This was corroborated by species richness indicating the numbers of the species (Figure 4b) and Faith’s phylogenetic distance metrics indicating community relatedness measurements (Figure 4c). For BR1, alpha diversity was largely stable from the transverse to the descending colon, while for BR2 and BR3, diversity continued to increase in the distal progression along the representative colonic regions for overall richness and Faith’s phylogenetic distance metrics. Taken together, this indicated that the influence of LGG on the overall microbial community alpha diversity was minimal and temporary and any impacts are secondary to larger inter-individual and regional differences.

The examination of differences in the overall microbial community composition (beta diversity) showed a similar relationship among the replicates, regions, and time points as observed in the alpha diversity metrics. Despite inter-individual differences, the AC communities are more similar to each other than to the TC and DC samples from the same bioreplicates. The TC and DC regions from BR1 cluster tightly together. A similar pattern was observed for BR2; however, for BR3, the TC and DC regions formed distinct clusters. For BR2 and BR3, the AC communities formed a single cluster as did the DC samples, indicating low inter-individual differences. This is true of the inoculum for BR2 and BR3 as well, as those communities are more similar to each other than to BR1 (Figure 4d, gray shapes).

The main taxa associated with the differences among the communities are shown in Figure 4d. TC and DC of BR1 were differentiated from BR2 and BR3 by the proportions of ASVs from the Bacteroidales order as opposed to members of the genera *Megasphaera* and *Acidaminococcus* (both of the Selenomonadales order). Interestingly, the abundances of closely related coliform genera *Escherichia* and *Citrobacter* distinguish between the AC of BR1 (*Escherichia*) and the TC and DC of BR2 and BR3, which were enriched in *Citrobacter*.

Notably, there was no clustering based on the time relative to LGG addition. This indicates that the overall microbial community structures were not altered by the addition of LGG, at least on the time scales covered by this experiment.

**Figure 5:**
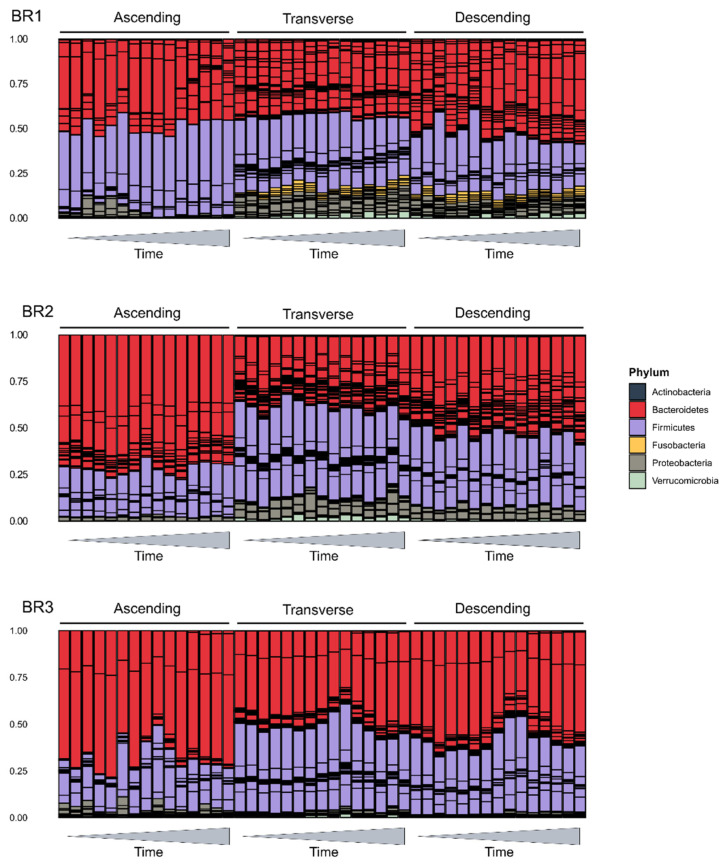
Relative abundance phylum barplots. Barplots showing relative abundances of the six top phyla in the SHIME samples. Phyla are shown as colors, and bars within each color represent individual taxa within that phylum. *X*-axis time indicates time since inoculation with LGG.

At a broader level, the bacterial phyla of Bacteroidetes and Firmicutes and the ratio of their abundances (F/B ratio) have been considered a marker for host health [40]. We examined the phylum level composition of the gut microbial communities to look at this ratio and the overall makeup of the communities (Figure 4e). All communities were dominated by members of the Firmicutes and Bacteroidetes, with substantially lower additional representation of Proteobacteria, Fusobacteria, Actinobacteria, and Verrucomicrobia (Figure 5). However, these communities varied in their overall composition, which was expected due to inter-individual variation in the gut microbiome. For example, BR1 had a greater relative abundance of Fusobacteria than BR2 and BR3, and Proteobacteria was more prevalent in BR1 and BR2 than BR3, which was in agreement with the Shannon’s diversity index measurements shown in Figure 4a. In agreement with the alpha-diversity metrics, this two-phylum dominance was more marked in the AC region in all bioreplicates, with diversity increasing in the TC and DC regions. The proportion of Bacteroidetes decreases distally along the GIT representative regions, with a corresponding increase in the F/B ratio. Only BR3 samples had significantly different F/B ratios by time points relative to LGG addition; however, the highest F/B ratios were all in samples from the time period immediately following LGG addition to the system, suggesting that LGG addition may favor beneficial, short-term, taxon-specific remodeling.

### 2.4. Impact of LGG Addition on SCFA Synthesis

**Figure 6:**
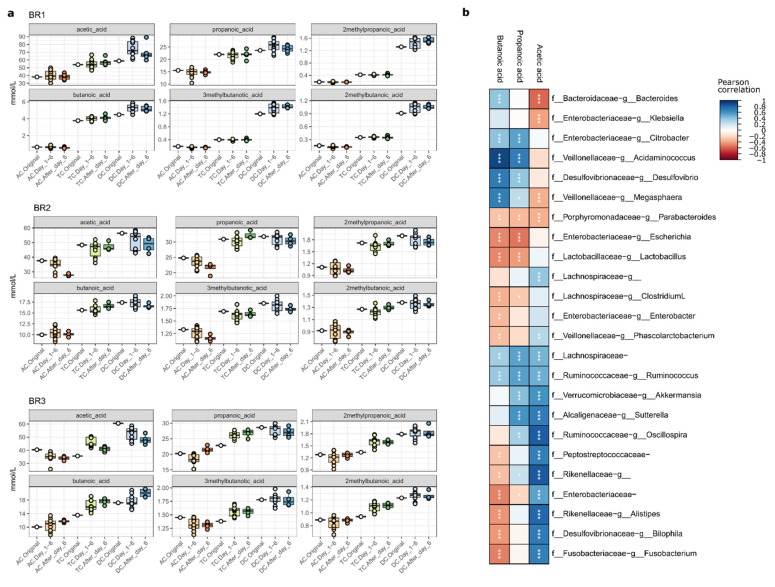
Short-chain fatty acid analysis. Concentrations of key SCFAs correlate with specific taxa. (**a**) SCFA concentrations generally increase along the reactor system, but do not always increase following LGG addition in individual reactors. Boxplots show IQR and median, with individual samples shown as points. (**b**) Pearson correlation between taxon relative abundance (ASVs summed at genus level) and three key SCFAs. Significant correlations are shown with asterisks. * *p* < 0.05; ** *p* < 0.01; *** *p* < 0.001.

We investigated the impact of LGG on SCFA production in gut microbial communities (Figure 6a,b). Comparing the three bioreplicates, the SCFA contents measured from BR2 and BR3 were higher than from BR1. Prior to LGG addition, the SCFA concentrations of the three bioreplicates increased along the reactor sequence from AC to TC, then to DC in a manner consistent with our previous finding [32]. While there were no significant differences in the concentrations of the measured SCFA following the addition of LGG, for some samples, the concentrations peaked in the immediate timeframe following LGG addition. This can be observed as greater variance in the “Day_1–6” sample groups seen in Figure 6. The addition of LGG to the three bioreplicates had no significant effect on this trend, despite a slight increase that was detected for 2M-/3M- butanoic acid and 2M-propanoic acid in the TC region (Figure 6a) which could potentially be attributed to the abundance of *F. Acidaminococcus* and *P. Suttrella* [41,42]. By comparing the 324 pairs of single SCFA amounts in the original community to the amount measured on days 8 and 10 post-LGG treatment, only 14 pairs showed a statistically significant difference, indicating that the impact of LGG on SCFA levels was minor and temporary, as it was gradually removed from the system. The Pearson correlation of SCFAs with taxon relative abundance (Figure 6b) illustrates the expected positive correlation between butanoic acid and key taxa such as *Acidaminococcus* and *Sutterella*, as well as *Bacteroides* and *Ruminococcus*. Given that SCFAs are readily consumed to support microbial metabolism, it is not surprising that large changes in SCFAs are not measurable following the addition of LGG because they are likely immediately consumed.

Conversely, an increase in a particular SCFA may be detected as a downstream increase in the abundance of a consumer of that metabolite. This underscores the subtle and community-dependent nature of LGG effects on the gut microbiome, which are likely mediated via metabolic cross-feeding and other community-level effects [43,44,45]. Such subtlety is valuable in a probiotic as dramatic shifts in gut microbial community composition or metabolic output could have undesirable effects on the human host.

## 3. Materials and Methods

### 3.1. Materials

Fecal homogenates from three donors were purchased from OpenBiome (Cambridge, MA, USA). According to the provider, the samples were collected using standard procedures from three anonymous individuals, aged 21–45, who consumed a normal Western diet, had an average BMI, were at least one-year antibiotic-free, and passed a screening similar to that used for blood donation [32]. LGG ATCC 53103 was purchased from American Type Culture Collection (Manassas, VA, USA). Frozen LGG stock was cultured anaerobically overnight in de Man, Rogosa and Sharpe broth (MRS) was purchased from Millipore Sigma (Burlington, MA, USA). Defined media for gut microbe growth and mucin carriers were purchased from ProDigest (Ghent, Belgium). Bile salts were obtained from Becton, Dickinson, and Company (Franklin Lakes, NJ, USA). Pancreatin and other chemicals of analytical grade were obtained from Sigma-Aldrich (Saint Louis, MO, USA).

### 3.2. Establishment of a Stable Gut Microbial Community in SHIME^®^

The in vitro LGG growth was tested using the SHIME^®^ platform (ProDigest, Ghent, Belgium), a computer-controlled artificial simulator resembling the human gastrointestinal tract (GIT). It consists of a series of bioreactors connected in sequence representing the different regions and phases of the GIT, specifically including the ascending colon (AC), transverse colon (TC), and descending colon (DC) regions. The SHIME^®^ was configured and operated with settings mimicking human intestinal retention time, pH, feeding cycle, temperature, and anaerobic conditions [32,33,34]. The experiment was conducted in triplicate by inoculating the SHIME with three fecal homogenates from three donors (representing 3 biological replicates, named BR1, BR2, and BR3, respectively) at 5% of the reactor volume. After inoculation, the system was fed three times daily with a defined medium (DM; ProDigest, Belgium) as the source of nutrients and pancreatic juice (PJ) containing pancreatin and bile acids to represent pancreatic and biliary secretions [32,46]. The experiments were run over 4–5 weeks (w), with a 2–3 w stabilization and control phase, and a 2 w experimental phase. Twice a week, 30 min before the first feeding cycle of the day, samples were taken from both the luminal and mucin phases of the bioreactors representing each of the three colon regions. Bacteria harvested in pellet form and bacteria-free supernatant (BFS) were stored at −80 °C for DNA extraction and short-chain fatty acid (SCFA) analysis, respectively.

### 3.3. Culturing LGG for Inoculation

LGG stock was thawed and cultured anaerobically overnight in de Man, Rogosa, and Sharpe broth (MRS) twice to ensure viability. The LGG was then diluted to a concentration of 0.5 McFarland units above baseline as determined using a densitometer (DEN-1; Thomas Scientific, Swedesboro, NJ, USA). Then, 20 mL of the diluted cultured was added to 1 L of MRS broth, gently shaken at 125 rpm for 10 h at 37 °C, and grown anaerobically to mid-exponential phase (concentration of 2.5–5 × 10^8^ CFU/mL). The culture was then centrifuged at 5000× *g* for 10 m at 4 °C. The pellet was resuspended in 25 mL of saline (0.9% NaCl) and used for inoculation of the SHIME’s AC region. The total amount of LGG added was approximately 2.5–5 × 10^9–11^ CFU, which resulted in a concentration of approximately 5 × 10^6–8^ CFU/mL.

### 3.4. Nucleotide Analysis

#### 3.4.1. DNA Extraction

DNA was extracted from pellets obtained from each bioreactor at each time point using the PowerSoil DNA extraction kit following the manufacturer’s guidelines (Qiagen, Hilden, Germany). The DNA concentration in each sample was quantified using a nanodrop (ThermoFisher, Waltham, MA, MA) and stored at −80 °C for LGG quantitation via qPCR and community analysis using 16s rRNA gene sequencing.

#### 3.4.2. Real-Time Quantitative Polymerase Chain Reaction (qRT-PCR) Detection of LGG

Levels of LGG were detected by quantitative PCR using a Taqman assay in a Roche Lightcycler 96 (Basel, Switzerland) following a previously published protocol [47]. Primers were purchased from Integrated DNA Technologies (Coralville, IA, USA) and used at a concentration of 1 pmol/μL. The forward primer sequence was 5′-CCGATCAACAGGCTCAGTGA-3′ and the reverse primer sequence was 5′-CATGTTGTGCGCTTGGAAAA-3′. The probe was purchased from Applied Biosystems (Foster City, CA, USA) and used at a final concentration of 0.05 pmol/μL. The probe sequence was 5′-TTGCACTTGATTGTTTCG-3′ and was 5′ end-tagged with 6FAM and 3′ end-tagged with MGBNFQ. FastStart Essential DNA Probes Master mix was purchased from Roche (Basel, Switzerland). The cycle program was set for initial denaturation at 95 °C for 600 s, followed by 45 cycles at 95 °C for 15 s and 60 °C for 60 s, and ended with a melting curve. Quantification of LGG in each sample was performed using the Roche Lightcycler 96 software (Basel, Switzerland) in comparison to an LGG standard. The standard consisted of LGG chromosomal DNA, and number of copies calculated using the following formula: [ng DNA × 6.0221 × 10^23^ molecules/mole]/[(3,010,111 × 660 g/mole) × 1 × 10^9^ ng/g].

#### 3.4.3. DNA Sequencing and Bioinformatics Analysis

DNA sequencing was performed at the Microbiome Center, Children’s Hospital of Philadelphia (CHOP). For 16S rRNA gene sequencing, the libraries were generated from DNA extracts using barcoded PCR primers targeting the V1V2 region of the bacterial 16S rRNA gene. The amplicons thus obtained were then sequenced on an Illumina Miseq a 2 × 250 bp reagent kit following the guidelines of the manufacturer (San Diego, CA, USA). DNA-free water and extraction blanks were used as negative controls, and 16S gene fragments of known amounts were used as the positive control. After demultiplexing and quality filtering, the read pairs were merged to form the exact V1V2 sequence using DADA2 software [48] with taxonomy assigned using the Green Genes database, version 13.8 [49]. The unique sequences were aligned using MAFFT [50], and a phylogenetic tree was built using FastTree [51] in the R environment. Weighted and unweighted UniFrac Distances were calculated using QIIME2 (VERSION). Additional analyses and visualizations were conducted in R (v 4.1.3) using the phyloseq, vegan, tidyverse, ape, picante, cowplot, reshape, RColorBrewer, ggplot2, and microViz packages. Alpha-diversity (Richness, Shannon index, and Faith’s phylogenetic diversity) metrics were calculated using an unfiltered feature table, and all other analyses were conducted post-filtering to remove singleton sequences. Significant intergroup differences in alpha diversity were identified using ANOVA with Tukey HSD (“honestly significant difference”) post hoc testing.

### 3.5. Short Chain Fatty Acids Analysis

Gas chromatography-mass spectrometry (GC-MS, Shimadzu QP2010 Ultra; Shimadzu, Columbia, MD, USA) equipped with a Stabilwax-DA column (0.25 mm × 30 m, 0.25 μm; Restek Corporation, Bellefonte, PA, USA) was used for SCFA analysis following a previously published protocol [32]. Linear SCFA, acetic acid, propionic acid, butyric acid, valeric acid, and caproic acid; as well as branched SCFA, 2-methylpropanoic acid, 3-methylbutanoic acid, 4-methylpentanoic acid, and 2-methyl hexanoic acid were analyzed in this study.

Samples collected from each time point in triplicate were thawed at ambient temperatures for 10 min. Then, a fraction of 1 mL from each sample was filtered, extracted three times with diethyl ether (1:1, *v*/*v*), and each extraction was measured three times on the GC-MS at the following settings: the initial column temperature was 125 °C for 1 min (m) and it increased to 250 °C at 10 °C/m followed by holding at this temperature for 3 m; injection volume was 1 µm; the split ratio was 1:20; and the helium flow rate was 1.0 mL/m. The interface temperature between the GC and MS was held at 250 °C and that for the ion source of the MS was 220 °C. Standard curves were constructed using individual SCFA standards at concentrations ranging from 5 to 2500 ppm and 2-methyl hexanoic acid as the internal standard.

## 4. Conclusions

In this research, we investigated the survival and growth profile of a probiotic strain LGG in three mature gut microbial communities developed within the SHIME using the fecal samples donated from three healthy Western diet consumers. Through this work, we found that the SHIME is an appropriate model for the examination of the persistence of LGG in vitro in a host gut microbial community. The methodology outlined above may also be suitable for the study of other probiotics. The introduction of LGG only slightly modified the Firmicutes/Bacteroides ratio but had no other appreciable impact on the gut microbial structures of the host communities. The survival, persistence, and growth profile of added LGG in the gastrointestinal tract is case-dependent but is at least 10 days for all tested cases.

## Data Availability

Raw sequencing reads have been deposited in the NCBI Sequence Read Archive under BioProject accession number PRJNA893635. SCFA and qPCR data can be found in the Supplementary Materials.

## Supplementary Materials

The following supporting information can be downloaded at: https://www.mdpi.com/article/10.3390/ijms232112973/s1.
